# A comparison of extended spectrum β-lactamase producing Escherichia coli from clinical, recreational water and wastewater samples associated in time and location

**DOI:** 10.1371/journal.pone.0186576

**Published:** 2017-10-17

**Authors:** Silje B. Jørgensen, Arne V. Søraas, Lotte S. Arnesen, Truls M. Leegaard, Arnfinn Sundsfjord, Pål A. Jenum

**Affiliations:** 1 Section for Medical Microbiology, Department of Laboratory Medicine, Vestre Viken Hospital Trust, Bærum, Norway; 2 Department of Clinical Microbiology and Infection Control, Akershus University Hospital, Lørenskog, Norway; 3 Norwegian University of Life Sciences, Oslo, Norway; 4 Institute of Clinical Medicine, University of Oslo, Oslo, Norway; 5 Norwegian National Advisory Unit on Detection of Antimicrobial Resistance, Department of Microbiology and Infection Control, University Hospital of North Norway, Tromsø, Norway; 6 Research Group for Host-Microbe Interactions, Department of Medical Biology, Faculty of Health Sciences, University of Tromsø, Tromsø, Norway; Natural Environment Research Council, UNITED KINGDOM

## Abstract

Extended spectrum β-lactamase producing *Escherichia coli* (ESBL-EC) are excreted via effluents and sewage into the environment where they can re-contaminate humans and animals. The aim of this observational study was to detect and quantify ESBL-EC in recreational water and wastewater, and perform a genetic and phenotypic comparative analysis of the environmental strains with geographically associated human urinary ESBL-EC. Recreational fresh- and saltwater samples from four different beaches and wastewater samples from a nearby sewage plant were filtered and cultured on differential and ESBL-selective media. After antimicrobial susceptibility testing and multi-locus variable number of tandem repeats assay (MLVA), selected ESBL-EC strains from recreational water were characterized by whole genome sequencing (WGS) and compared to wastewater and human urine isolates from people living in the same area. We detected ESBL-EC in recreational water samples on 8/20 occasions (40%), representing all sites. The ratio of ESBL-EC to total number of *E*. *coli* colony forming units varied from 0 to 3.8%. ESBL-EC were present in all wastewater samples in ratios of 0.56–0.75%. ST131 was most prevalent in urine and wastewater samples, while ST10 dominated in water samples. Eight STs and identical ESBL-EC MLVA-types were detected in all compartments. Clinical ESBL-EC isolates were more likely to be multidrug-resistant (p<0.001).

This study confirms that ESBL-EC, including those that are capable of causing human infection, are present in recreational waters where there is a potential for human exposure and subsequent gut colonisation and infection in bathers. Multidrug-resistant *E*. *coli* strains are present in urban aquatic environments even in countries where antibiotic consumption in both humans and animals is highly restricted.

## Introduction

Antimicrobial resistance (AMR) is an increasing worldwide problem that affects both humans and animals. The fear that a post-antibiotic era is approaching enforces us to embrace the One Health perspective, in the recognition that human health is closely related to the health of animals and to the environment [1, 2].

Extended spectrum β-lactamase producing *Escherichia coli* (ESBL-EC) are transferred between humans and animals, but may also spread in aquatic environments and potentially contaminate and infect exposed individuals [2, 3]. ESBL-EC are common in farm and companion animals in many parts of the world [4, 5]. There are also numerous reports of their occurrence in wild-life [6–9] and the environment on several continents [5, 10–16]. Also in the Scandinavian countries, ESBL-EC is an increasing problem in human medicine, and their presence has been described in domesticated animals and livestock [17–21], as well as in the red fox [22], in gulls [6], and in potable water [11].

There are few reports using spatial and temporal methods to explore possible AMR transmission between the environment and humans. We have previously shown that people with community acquired urinary tract infection were more likely to be infected with an ESBL-EC if they had been bathing in freshwater within the last 12 months [23]. To further investigate possible transmission routes, whole genome sequencing (WGS) was used to compare ESBL- EC isolates from freshwater lakes, saltwater basins and a nearby wastewater treatment plant with human clinical strains from people living in the same area.

### Ethics statement

The study was approved by the Regional Committee for Medical and Health Research Ethics December 11^th^ 2008 (reference number: 2009/2037 BS-08901b). It was funded by South-Eastern Norway Regional Health Authority (grant number 2013060), and is registered in the ClinicalTrials.gov registration system, registration ID NCT01838213, 2013. The included human population has been part of three previously published studies [23–25], and some of the environmental samples have been mentioned in a short communication in press [26].

## Materials and methods

### Collection of samples and bacterial culture

Bacterial samples were obtained from three compartments: recreational water, wastewater and human urine. Recreational water samples were collected on five different dates during the summer season (May-September 2010) at one freshwater and three saltwater beaches located in our laboratory’s catchment area close to the Norwegian capital Oslo. Sterile containers of 1 liter were rinsed three times at the sampling site before the first sample was collected from a depth of 0.5–1 meter. Portions containing 10–500 ml of water were vacuum-filtered. The filters were subsequently grown on Brilliance® agar (Oxoid, Basingstoke, UK) and ChromeID® ESBL plates (BioMérieux, Marcy-l’Etoile, France) at 35°C for 40–48 hours. Purple colonies on Brilliance plates were counted and considered an estimate for the total of *E*. *coli* colony forming units (CFUs). Individual purple colonies on ChromeID ESBL plates representing potential ESBL-EC isolates were frozen for later analysis. This method allowed us to estimate the total amount of *E*. *coli*, and calculate a CFU-ratio of ESBL-EC/total *E*. *coli*. The recreational water quality regarding *E*. *coli* content was assessed according to the EU bathing water directive (Directive 2006/7/EC), where the water quality is described as excellent, good, sufficient, or not-sufficient with different limits of allowed amount of *E*. *coli* in freshwater and seawater.

Daily flow proportional influent wastewater samples were collected from our local wastewater treatment plant (Vestfjorden Avløpsselskap) on the same days as the environmental samples. The samples were transported in sterile containers, filtered and grown on Brilliance® and ChromID ESBL plates, like the water samples. As the concentration of bacteria was considerably higher in this material, quantities of 1, 5, 10 and 50 μl of water were plated directly in this procedure. All purple colonies from ChromID ESBL plates were individually frozen for later analysis.

**Fig 1:**
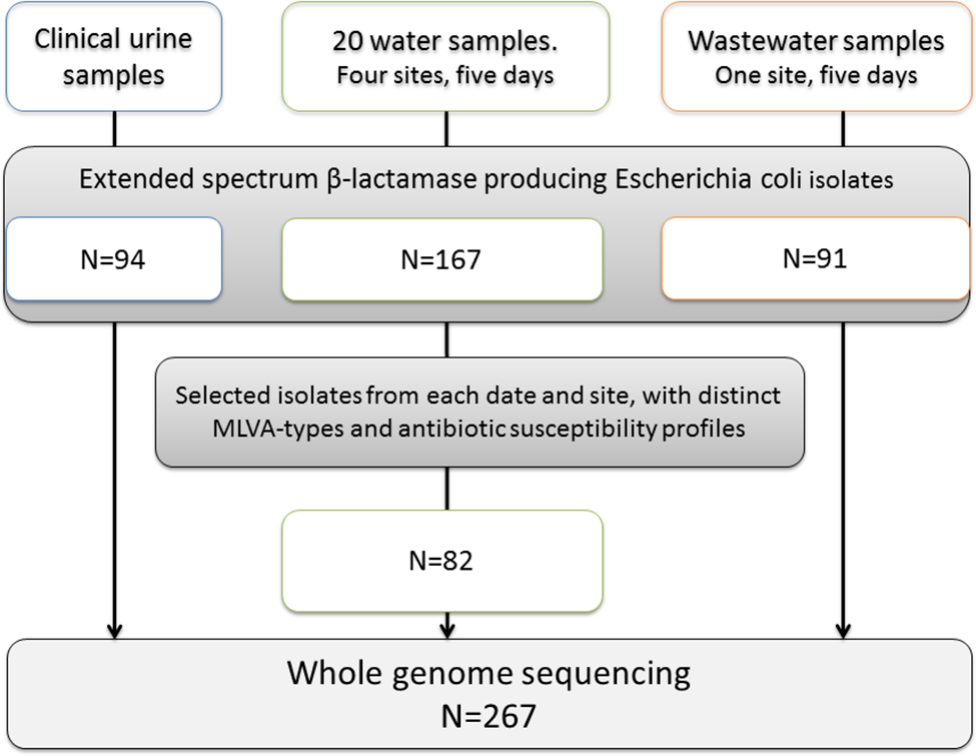
Sample selection chart.

Urinary samples from people living in the catchment area of the wastewater plant, and with close proximity to the recreational water sites, were collected between February 2009 and April 2011. The cohort of 94 Norwegian patients with community acquired urinary tract infection caused by ESBL-EC has been described previously [24]. Written consent was obtained from all participants. A sample selection chart is presented in Fig 1.

### ESBL identification and susceptibility testing

Antibiotic susceptibility testing (AST) and bacterial identification were performed using ChromID ESBL agar, VITEK-2 system (BioMérieux, Marcy l’Etoile, France) and MALDI-TOF (Bruker Daltonics, Bremen, Germany). Non-susceptible isolates were defined as isolates either classified as intermediate or resistant to the investigated drugs. The drugs selected for AST were the drugs routinely used in treatment of urinary tract infections according to Norwegian guidelines (https://unn.no/fag-og-forskning/arbeidsgruppen-for-antibiotikasporsmal-og-metoder-for-resistensbestemmelse-afa#resistenspaneler). AST interpretations were according to EUCAST (http://www.eucast.org). ESBL-confirmation was performed by E-test (AB-Biodisk, BioMérieux). The ChromID ESBL plates were read after 18 hours and re-examined after 48 hours, because we wanted the detection method to be as sensitive as possible. All isolates growing on the selective medium were tested by ESBL E-test and a multiplex PCR for detection of bla_CTX-M_ [27]. If they were resistant to cefotaxime and/or ceftazidime, but negative in the bla_CTX-M_ PCR, they were investigated by Microarray Check-MDR CT101 (Check-Points B.V., Wageningen, the Netherlands) targeting bla_TEM_, bla_SHV_, bla_NDM_, bla_KPC_, as well as the following plasmid mediated AmpC variants: CMY, DHA, FOX, ACC, ACT, MIR and MOX. Only isolates positive for ESBLs (CTX-M, TEM, SHV) or plasmid mediated AmpC were included in further analyses. Colistin resistance was examined by the broth microdilution method using in-house designed premade Sensititre microtiter plates (TREK Diagnostics, Cleveland, OH, USA) for *mcr-1* positive strains.

### MLVA typing

Clonal relatedness between isolates was examined by a multilocus variable number of tandem repeats assay (MLVA) [28]. Briefly, PCR-products were subjected to capillary electrophoresis, and each peak was identified according to colour and size by GeneMapper software (Applied Biosystems, Foster City, CA, USA). Allele numbers were assigned according to fragment sizes [28]. Character values were entered into BioNumerics (Applied Maths, Sint-Martens-Letem, Belgium), and dendrograms were constructed using categorical coefficients and the Ward algorithm. A standard minimum spanning tree was generated using categorical coefficients together with the single and double locus variance priority rules.

### Whole genome sequencing

All urine and wastewater isolates were sequenced on the Illumina HiSeq platform (Illumina, San Diego, USA) generating 150 base pairs paired-end reads. Isolates from recreational water were selected for WGS as follows: From each day and each site, all isolates were sequenced unless there were multiple isolates with identical MLVA-type and AST-pattern. In such cases, only one of these isolates was sequenced. Assembly, determination of resistance genes, plasmids, virulence factors and multi locus sequence types (STs) was performed by using online tools (ResFinder [29], PlasmidFinder [30], VirulenceFinder[31]) from Center for Genomic Epidemiology, Technical University of Denmark, (https://cge.cbs.dtu.dk/services) [32]. The settings for the online search tools are described in supplementary file S2.

### Statistical analyses

PASW statistics software, version 21.0 (IBM SPSS, IL, USA) were used for statistical analyses. Univariate and multivariate analyses were done by binary logistic regression, and p-values ≤ 0.05 were considered significant.

## Results

### ESBL detection in recreational water and wastewater

*E*. *coli* were detected in all samples. Eight out of 20 recreational water samples (40%) contained ESBL-EC, and ESBL-EC were present at all sampling sites in an average concentration of 0.76 CFU/100 ml. ESBL-EC were found on four out of five occasions at the freshwater location. AST and MLVA were performed on all isolates from recreational water (N = 167) (S1 Table), and 82 unique isolates were selected for WGS (Fig 1). All wastewater samples were ESBL-EC positive, and 91 confirmed ESBL-EC isolates were retrieved from this compartment. These were all included in the WGS analyses. The CFU-ratio of ESBL-EC/total *E*. *coli* in recreational water and wastewater samples varied between 0–3.8% and 0.56–0.75%, respectively (Table 1).

**Table 1:** ESBL producing *E*. *coli*/total amount of *E*. *coli* per date and location in recreational water and wastewater samples.

	ESBL producing *E*. *coli* CFU^1^/estimated number of *E*. *coli* CFU per 1 L water (%)									
	May 19^th^		June 14^th^		June 22^nd^		Aug. 16^th^		Sept. 15^th^	
Freshwater	6/1160	(0.52)	1/520	(0.19)	0/30	(0)	4/2,100	(0.19)	8/1,070	(0.75)
Saltwater 1	0/3000	(0)	0/30	(0)	0/500	(0)	30/800	(3.8)	72/12,100	(0.60)
Saltwater 2	0/1850	(0)	0/240	(0)	0/30	(0)	26/3,800	(0.68)	0/180	(0)
Saltwater 3	0/100	(0)	0/50	(0)	0/10	(0)	0/100	(0)	4/690	(0.60)
Wastewater	320 ESBL-EC^2^		120/16,000	(0.75)	380/57,000	(0.67)	320/49,000	(0.65)	150/27,000	(0.56)

^1^Colony forming units

^2^ Missing data on total *E*. *coli* CFU

### Antimicrobial susceptibility profiles

Comparison of antibiotic susceptibility profiles were made between the urine strains (n = 94), the wastewater strains (n = 91) and those of the recreational water strains included in the genotypic resistance analyses (n = 82). The non-susceptibility rates towards quinolones, gentamicin, aztreonam, trimethoprim and trimethoprim/sulfamethoxazole were significantly higher in urine strains compared to recreational water strains. Non-susceptibility rates towards quinolones, ceftazidime and aztreonam were also higher in urine than in wastewater strains (Table 2). Multidrug-resistance (non-susceptibility towards two or more antibiotic classes other than β-lactams) was most frequent in urinary strains (odds ratio (OR) 5.9 compared to recreational water strains, confidence interval (CI) 2.7–12.8, p<0.001, OR 3.5 compared to wastewater strains CI 1.6–7.5, p <0.001). The differences in multidrug-resistance were still significant when adjusted for the high number of ST131 among the clinical isolates.

**Table 2:** Phenotypic non-susceptibility of ESBL producing *E*. *coli* from recreational water (N = 82) and wastewater (N = 91) compared to strains from urine (N = 94).

	Urine %	Water %	p	Wastewater %	p
**Ampicillin**	100	100	1	99^1^	1
**Cefuroxime**	100	99^1^	1	99^1^	1
**Cefotaxime**	99	98	1	96	0.21
**Aztreonam**	98	85	0.03	85	0.007
**Ceftazidime**	85	77	0.06	66	0.001
**Trimethoprim**	77	51	0.002	66	0.09
**Trimethoprim-sulfamethoxazole**	72	50	0.008	62	0.1
**Ciprofloxacin**	69	35	0.001	42	0.001
**Gentamicin**	34	20	0.03	34	0.8
**Mecillinam**	3	0	1	6	0.5
**Piperacillin-tazobactam**	5	5	0.48	7	1
**Nitrofurantoin**	1	0	1	7	0.09
**Meropenem**	0	0	-	0	-
**Multidrug-resistance**^**2**^	88	52	0.001	68	0.001

^1^100% non-susceptibility would be expected as the isolates initially grew on selective media, and contain ESBLs according to initial tests and DNA-analysis. The wastewater isolate which was susceptible to ampicillin and cefuroxime contained *bla*_*CMY-58*_. The isolates from recreational water which were susceptible to cefuroxime both contained *bla*_*SHV-12*_.

^2^Resistance to ≥ 2 different antibiotic classes other than β-lactams.

### β-lactamases and co-resistance

Regardless of sample source, CTX-M-15 was the dominating ESBL, followed by CTX-M-14 (Table 3). Several strains contained multiple ESBLs, the most common combination being CTX-M and TEM, followed by CTX-M and OXA-1. Acquired resistance genes conferring resistance towards trimethoprim, macrolides, phenicols, quinolones and aminoglycosides were less frequent in recreational water strains compared to urinary strains (Table 4). The plasmid-born colistin resistance gene *mcr-1* was detected in two isolates from recreational water, as previously described [26]. The correspondence between AST-profiles and resistance determinants as detected by ResFinder was 77% for trimethoprim, 44% for gentamicin/aminoglycoside resistance and 59% for ciprofloxacine/fluoroquinolone resistance (*qnr* and *aac(6’)lb-cr* combined).

**Table 3:** Distribution of β-lactamases (*bla*) in *E*. *coli* isolates according to sample source.

Β-lactamase	Urine	Recreational water	Wastewater
	%	%	%
*bla*_CTX-M-1_	7	13	2
*bla*_CTX-M-2_	0	1	0
*bla*_CTX-M-3_	3	0	3
*bla*_CTX-M-3-like_	1	0	0
*bla*_CTX-M-8_	0	0	1
*bla*_CTX-M-9_	0	4	1
*bla*_CTX-M-14_	13	12	18
bla_CTX-M-14b_	4	0	3
*bla*_CTX-M-15_	52	48	45
*bla*_CTX-M-24_	3	0	1
*bla*_CTX-M-27_	9	1	5
*bla*_CTX-M-32_	1	0	1
bla_CTX-M-55_	1	6	4
*bla*_CTX-M-139_	0	0	1
*bla*_CMY2_	0	0	1
*bla*_CMY42_	0	0	1
*bla*_CMY-58_	0	0	1
*bla*_CMY-60_	1	0	0
*bla*_OXA-1_	29	5	22
*bla*_OXA-1-like_	0	0	1
*bla*_OXA10_	0	0	1
*bla*_SHV-12_	4	2	3
*bla*_TEM-1B_	56	44	31
*bla*_TEM-1B like_	5	9	9
*bla*_TEM-1C_	1	0	0
*bla*_TEM-1D-like_	0	5	0
*bla*_TEM-33-like_	0	1	1
*bla*_TEM-52B_	0	0	1
*bla*_TEM-52C_	0	1	0

**Table 4:** Comparison of the frequencies of acquired resistance genes in ESBL-*E*. *coli* isolates from urine strains versus isolates from recreational water and wastewater.

Resistance determinant	Urine N = 94		Recreational water N = 82		p	Wastewater N = 91		p
	N	%	N	%		N	%	
ESBL^1^ but no *bla*_CTX-M_ genes	5	5	2	2	0.34	8	9	0.36
Sulphonamide^2^	74	78	53	65	0.06	62	68	0.15
Trimethoprim^3^	69	73	39	48	0.001	60	66	0.36
Tetracycline^4^	69	73	54	66	0.35	60	66	0.35
Aminoglycoside^5^	62	65	55	67	0.08	71	78	0.91
Macrolide^6^	55	58	27	33	0.001	36	40	0.02
Phenicol^7^	39	41	14	17	0.001	34	35	0.67
Fluoroquinolone/aminoglycoside^8^	28	29	6	7	0.001	18	20	0.16
Fluoroquinolone^9^	2	2	3	4	0.55	9	10	0.04
Colistin^10^	0	0	2	2	1	0	0	-

^1^bla_SHV_, bla_CMY_

^2^sul-1, sul-1-like, sul-2, sul-2-like, sul-3

^3^dfrA1, dfrA5, dfrA7, dfrA12, dfr14-like, dfr17

^4^tet(A), tet(A)-like, tet(B)

^5^strA, strA-like, strB, strB-like, aadA1, aadA1-like, aadA2, aadA2-like, aadA12, aadA5c, aadA24-like, addB, aac(3)lla, aac(3)lla-like, acc(3)lld-like, aph(3’)-la, aph(3’)-la-like, aph(3’)-lc, aph(3’)-lc-like, aph(6)-lc-like

^6^erm(B)-like, mph(A), mph(A)-like

^7^catA1-like, catB3-like, cmlA1-like, floR-like

^8^aac(6’)lb-cr, aac(6’)lb-cr-like

^9^QnrB19, QnrS1, QnrS1-like

^10^mcr-1

### Detection of plasmid replicons and virulence factors

Many different plasmid replicons were present, and in various distributions (S2 Table). Briefly, the two dominating replicon types were IncF, present in 90% of the strains, and IncI1 present in 31%. No significant differences in IncF or IncI1 were observed between the different compartments.

A search for virulence factors associated with gastrointestinal *E*. *coli* infections (*eae*, *eaeA*, *bfp*, *stx1/vtx1*, *stx2/vtx2* and *ipaH)* revealed two strains with *eae*. These isolates were both retrieved from wastewater. One of them was ST3 and contained *bla*_CTX-M-15_ while the other was ST381 and contained *bla*_CMY-58_.

### Distribution of MLVA-types and STs

**Fig 2:**
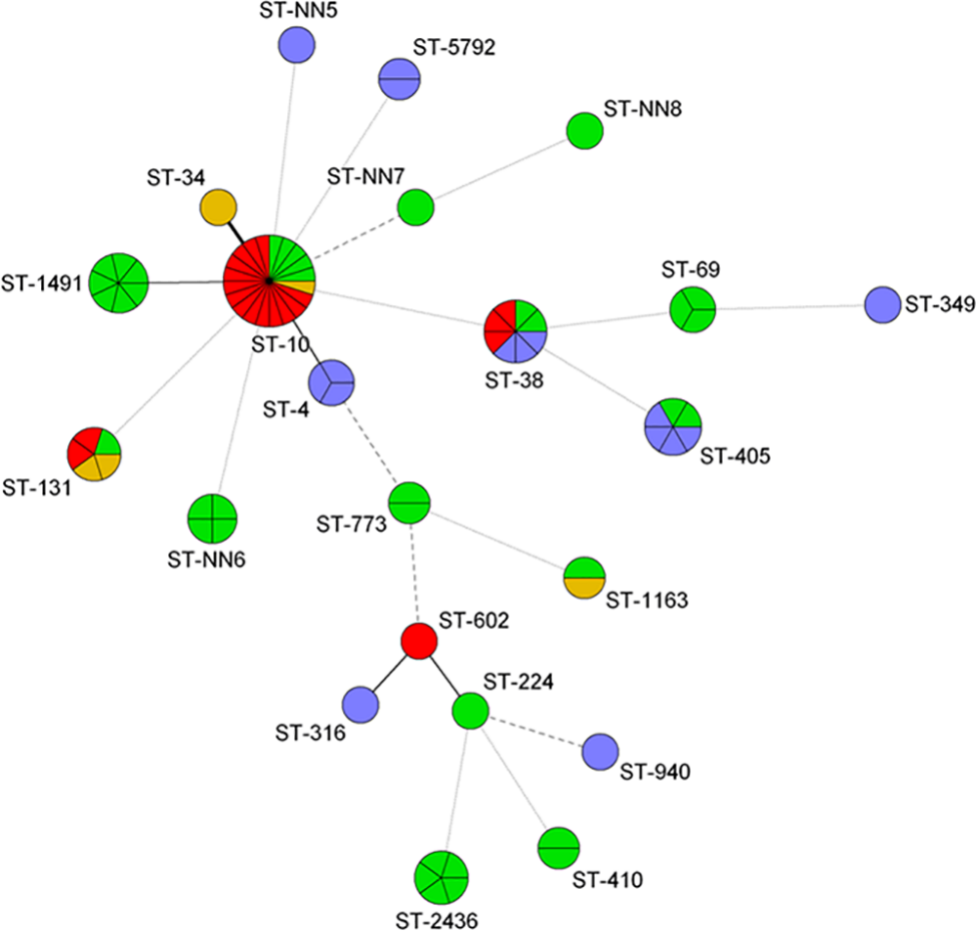
Minimum spanning tree of ESBL producing *E*. *coli* multi locus sequence types (STs) detected in recreational water at different sites. Blue, green, red and yellow colours represent isolates detected at Bondivann (freshwater), Kalvøya, Holmenskjæret and Furstranda, respectively. Each node represents one ST. The thickness and length of the lines vary according to the similarity of STs in the connected nodes.

**Fig 3:**
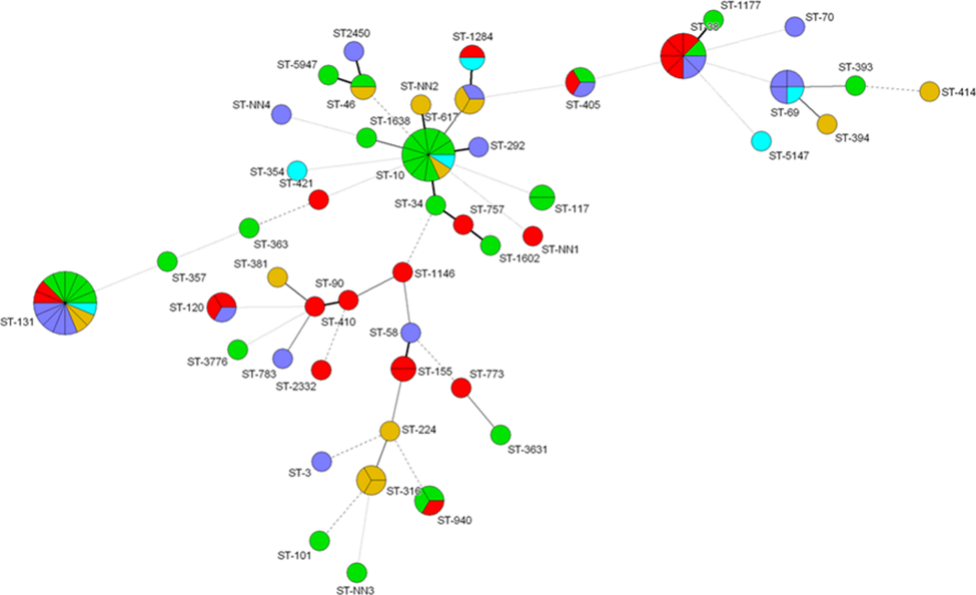
Minimum spanning tree of ESBL-producing *E*. *coli* (N = 91) from wastewater. Each node represents one ST, while each colour represents one of five sampling dates. The thickness and length of the lines vary according to the similarity of STs in the connected nodes. Red = 19-May-2010, blue = 14-June-2010, orange = 22-June-2010, green = 16-August-2010, purple = 15-September-2010.

**Fig 4:**
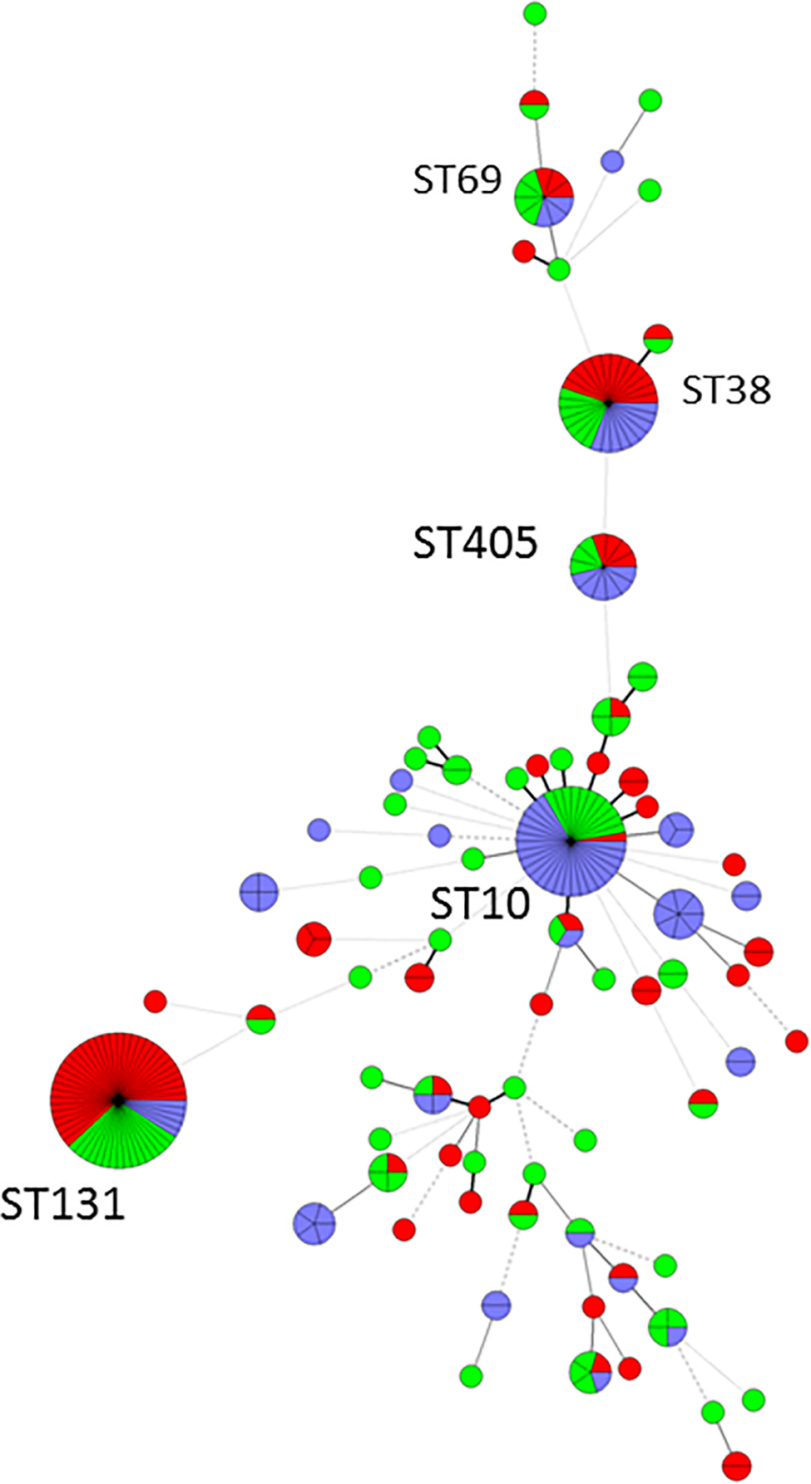
Minimum spanning tree of ESBL producing *E*. *coli* STs detected in recreational water (blue, N = 82), urine (green, N = 94) and wastewater (red, N = 91). Each node represents one ST. The thickness and length of the lines vary according to the similarity of STs in the connected nodes.

We detected 42 MLVA-types and 22 STs among ESBL-EC from recreational water. ST131, ST38 and ST10 were observed in three locations, and ST405 and ST1163 were found in two different locations (Fig 2). Only ST38 was found repeatedly on the same site. ST131, ST10 and ST38 were also the most frequent MLSTs in the wastewater samples, being present on five, three and three occasions respectively (Fig 3). ST131 and ST38 were present in urine samples from 35 (37%) and 13 patients (14%), respectively, and ST10 only in one. A minimum spanning tree of STs according to sample material is given in Fig 4 (Fig 4).

There was a large variation in STs, but eight STs were found in all sample materials; ST131, ST10, ST38, ST405, ST69, ST940, ST410, and ST34. There were several MLVA-types within each of these STs (S1 File). Some of the MLVA-types contained isolates from different sources. Within one MLVA-type of ST38, the β-lactamases and plasmid replicons were the same in all isolates from urine, wastewater and recreational water. Otherwise, most MLVA-types contained isolates with different β-lactamases and plasmid replicons.

ST10, the most frequent ST in recreational water samples, consisted of 36 isolates dispersed in 14 different MLVA-types. S1 Fig illustrates how the MLVA-types of ST10 strains were distributed in the different compartments (S1 Fig).

## Discussion

To explore whether recreational water is a possible source for ESBL-EC colonisation and infection in humans, we have identified and compared ESBL-EC from urine, recreational water and wastewater samples associated in time and geographical location. So far, studies of this sort have been lacking [2]. We show that ESBL-EC are readily detected in wastewater, and are frequently present in recreational water. However, they constitute only a relatively small portion of total *E*. *coli* in both environments (3.8% or less). The three compartments represent highly similar populations of ESBL-EC, with some significant differences.

The general amount of *E*. *coli* in our samples increased during the summer season, and six out of eight samples containing ESBL-EC were retrieved on the two last sampling dates in August and September. It seems that detection of ESBL-EC is associated with high amounts of total *E*. *coli*, as previously described by others [33]. If the total amount of *E*. *coli* is very low, and the proportion of ESBL-EC is small, our methods are probably not sensitive enough for ESBL-EC to exceed the detection limits. Although studies on ESBL detection in various habitats are steadily increasing [2, 5, 11–13, 34, 35], there are still few studies describing not only the prevalence of ESBL-EC, but also the proportion of ESBL-EC compared to total *E*. *coli* in recreational water. In a similar study conducted in the Netherlands in 2011/2012, ESBL-EC represented 0–1% of total *E*. *coli*, and ESBL-EC were present in 62% of recreational water samples [33]. In a study from Croatia in 2009 to 2013, Maravic and co-workers conducted a study of recreational seawater. Here they found considerably higher proportions of ESBL-EC (7.7%), even though they used non-selective culture methods [14]. The isolates from recreational water were clonally related to clinical isolates. As ESBLs in clinical *E*. *coli* isolates are more common in Croatia than in Norway and the Netherlands [36], it is not very surprising that they are also more prevalent in Croatian environmental samples. However, it is also important to note that the general levels of *E*. *coli* were much higher in the Croatian samples. In summary, all three studies support our hypothesis that recreational water is a possible source of human exposure to ESBL-EC, and they illustrate that the levels of ESBL-EC vary in different locations. According to the EU bathing water directive (Directive 2006/7/EC), 17 samples in our study held the quality classified as “excellent”, while two samples qualified as “good” and one less than sufficient, regarding the amount of *E*. *coli* present in the samples. The number of coliform bacteria in recreational water may vary substantially according to water temperature, rainfall and other conditions [33, 37]. Hence, recording of weather data could have been relevant for the interpretation of our results. Also, our estimates of bacterial amounts could have been more accurate if more samples had been filtered and cultured in parallel.

We have only detected relatively small amounts of ESBL-EC in recreational water, and the risk of becoming colonized during bathing is difficult to estimate. Water-born outbreaks of pathogenic Shiga toxin-producing *E*. *coli* (STEC) have been reported earlier [38], demonstrating the potential risk of *E*. *coli* transmission to humans from recreational water. STEC strains can be ESBL-producers [39, 40]. Although no Shiga toxin-genes were detected in this study, we did find two strains with the *E*. *coli* attaching and effacing gene, *eae*, which is associated with enteropathogenic *E*. *coli* (EPEC) strains.

In a previous case-control study we identified recreational freshwater swimming as an independent risk factor for community acquired urinary tract infection caused by ESBL-EC [23]. Patients who had been swimming in freshwater during the last 12 months had an odds ratio of 2.1 of ESBL-EC compared to non-ESBL-EC (95% CI 1.0–4.3, p = 0.04). The ESBL-EC dose required for colonisation and/or infection after exposure to contaminated recreational water will probably depend on various strain and host factors. It is also known that antibiotic exposure reduces the gut colonisation resistance provided by a normal microbiota [41].

The ESBL-ECs we detected in wastewater are probably an approximation of the mix of commensal and pathogenic strains carried by humans in the catchment area, while the urine samples represent more virulent strains. One can assume that recreational water is contaminated by fecal strains from wild animals, live-stock and manure, as well as humans, and maybe also by wastewater [42]. The previously mentioned study from the Netherlands concludes that ESBL-EC strains in recreational water derive both from wastewater and other sources [33]. Because we have only examined the wastewater from one single wastewater treatment plant, it is difficult to say whether the amount of ESBL-EC at this plant is representative of other Norwegian plants.

In this study we used both MLST and MLVA to assess clonal relatedness between ESBL-EC isolates from different sources. We detected a large variation in STs, but eight STs were found in all compartments. The pandemic, pathogenic clone ST131 phylogroup B2 dominated in clinical samples (34/94), was frequent in the wastewater samples (16/91), and rare in the recreational water samples (5/82). ST38 phylogroup D was well represented in all sources, while ST10 phylogroup A dominated in water, but was only found in urine on one occasion. ST10 has previously been described as a successful ESBL-EC occurring in swine, cattle and poultry as well as in humans [4, 43]. It has also been detected in surface water and wastewater in Portugal [44], the Netherlands [33], the UK [34], from the river Danub [13], and in migrating penguins [9]. The presence of ESBL-EC ST10 in different water and wastewater sources on different dates, calls for further investigation of the dissemination of this strain in other ecological niches in Norway, such as farm animals, manure, birds and wild animals [9]. The other strains that we retrieved from all our sources (ST69, ST405 and ST410) are also well-known *E*. *coli* hosts of ESBLs [4, 45].

Comparisons of ESBL-EC AST patterns across the three compartments revealed that multidrug resistance is more common in urine strains even if we adjust for the dominance of ST131 in clinical strains. We also performed a comparative analysis of the correlation between the phenotypic susceptibility towards fluoroquinolones, trimethoprim and aminoglycosides and the presence of relevant resistance genes from WGS-data. WGS has the potential to predict antimicrobial susceptibility, but the available evidence for using WGS as a tool to infer antimicrobial susceptibility accurately is either poor or non-existent [46]. Resistance towards fluoroquinolones is conferred by plasmid-mediated *qnr-*genes or acetyltransferases, but chromosomal mutations in the *gyr*A or *par*C genes are the more common cause [47]. However, chromosomal resistance is not detected by the ResFinder software. This is reflected in our results; according to AST-profiles, many isolates are non-susceptible to ciprofloxacin even if we don’t detect any acquired fluoroquinolone resistance genes by ResFinder. There are available tools online for identification of SNPs conferring chromosomal resistance, e.g. the CARD database and Resistance Gene Identifier (www.card.mcmaster.ca), but extensive analysis of chromosomal resistance genes was not within the scope of this study. Resistance towards aminoglycoside can also be caused by various mechanisms. It is most often linked to modifying enzymes encoded by plasmids and transposons, but adaptive mechanisms, such as membrane protein changes and regulation of genes involved in the anaerobic respiratory pathway may also be involved [48]. We detected genes with a potential role in conferring aminoglycoside resistance in several isolates which are susceptible to gentamicin according to their AST-profile. This is not surprising, as many of the genes conferring aminoglycoside resistance are highly substrate specific [48]. They may confer resistance to other aminoglycosides, but not to gentamicin. Hence, the phenotypic susceptibility testing is an essential supplement to the genetic detection of acquired resistance genes, as appropriate antibiotic treatment is based on susceptibility.

We find identical or closely related MLVA-types across compartments, including a ST38 strain with identical MLVA-type, resistance genes and replicons in all three compartments. These observations underline the interplay between strains, resistance determinants and plasmids between human and recreational water reservoirs.

In this study, strains with different MLVA-types and AST-profiles were selected for WGS to emphasize strain diversity. Thus, the abundance of single STs in some samples is not reflected in the figures, but illustrated in S1 Table. When we analyzed the MLVA fingerprints within separate STs, we saw that even this high-resolution tool is not sensitive enough to identify isolates with identical genetic makeup in a setting like this, where isolates are collected from different compartments over a timespan of many months. With few exceptions, there are variations in plasmid and resistance genes content also within MLVA-types. Never the less, MLVA may serve as a simple and effective surrogate marker for detection of ST131 or ST38, as their MLVA-patterns in the first four loci seem quite conserved [49, 50].

## Conclusion

This study supports our hypothesis that humans can be exposed to potentially harmful ESBL-EC strains through recreational bathing. Even in Norway, where antibiotic consumption in both humans and animals is highly restricted, multidrug-resistant *E*. *coli* strains are readily detected in aquatic environments. However, multidrug-resistance is less frequent in strains from recreational water than in strains from wastewater and clinical urine samples.

## Supporting information

S1 Table E. coli MLVA-types in recreational water. (XLSX)

S2 Table Plasmid replicons. (XLSX)

S1 File Minimum spanning tree of ESBL producing E. coli MLVA-types in different STs. (PDF)

S2 File Search settings for ResFinder, PlasmidFinder and VirulenceFinder. (PDF)

S1 Fig Minimum spanning tree of ESBL producing E. coli ST10 MLVA-types. (TIF)
