# Supplementary material for: A comparison of extended spectrum β-lactamase producing Escherichia coli from clinical, recreational water and wastewater samples associated in time and location
Source: PLoS One. 2017 Oct 17;12(10):e0186576. doi: 10.1371/journal.pone.0186576 (PMC5645111; doi:10.1371/journal.pone.0186576)
Supplement: S1 File — (PDF) [file pone.0186576.s003.pdf]

Figure S1 Minimum spanning tree of ESBL producing *E. coli* MLVA-types in different STs. Each node represents one MLVA-type. The thickness and length of the lines vary according to the similarity of STs in the connected nodes. Green=urine, red=wastewater, purple=recreational water.

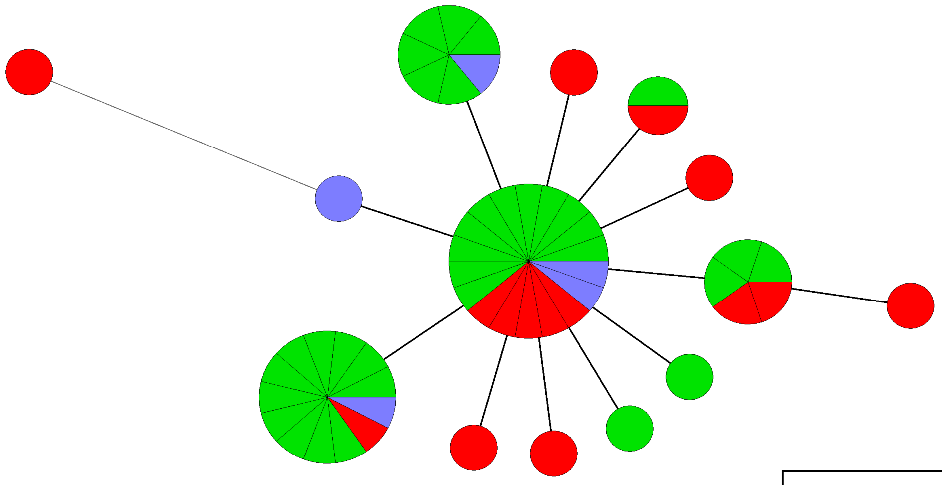

Fig S1A ST131

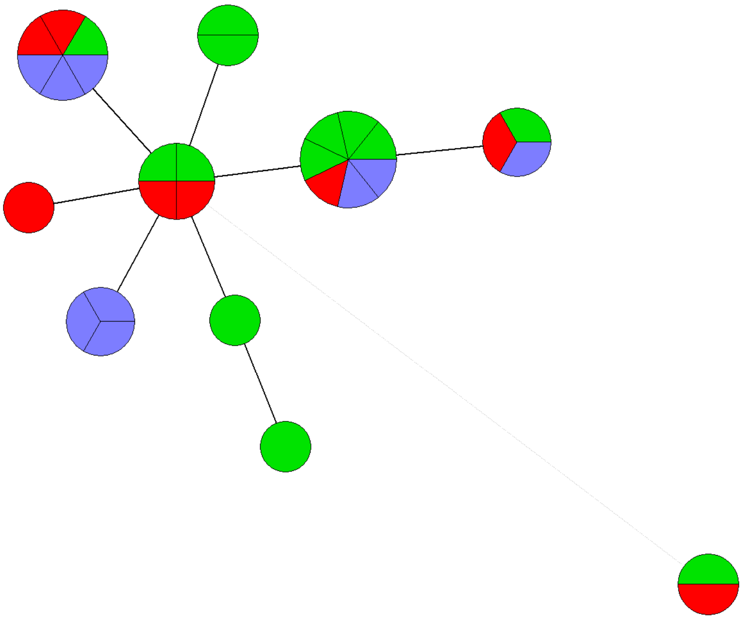

Fig S1B ST38

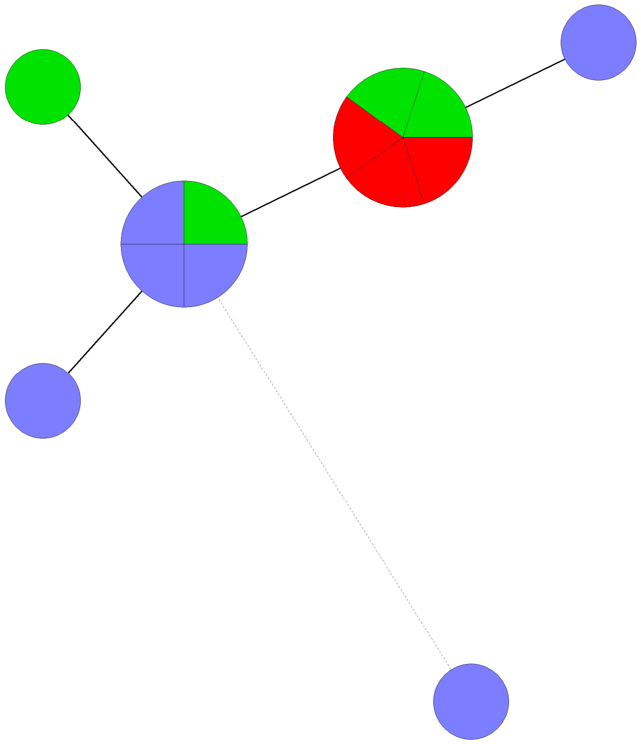

Fig S1C ST405

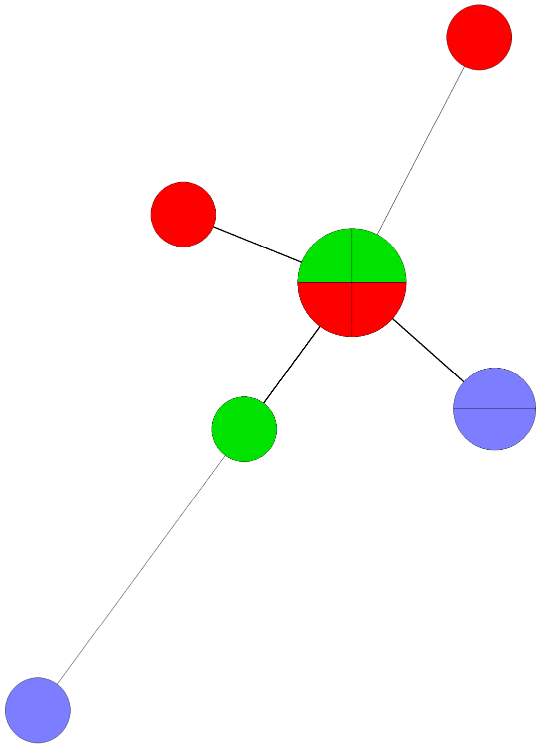

Fig S1D ST69

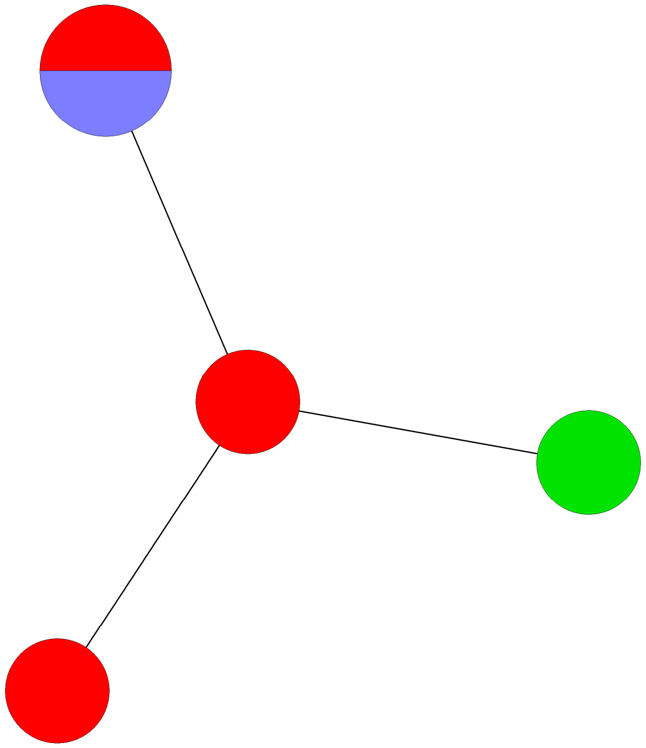

Fig S1E ST940

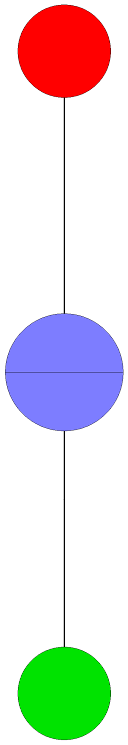

Fig S1 F ST410
