# Supplementary material for: A comparison of extended spectrum β-lactamase producing Escherichia coli from clinical, recreational water and wastewater samples associated in time and location
Source: PLoS One. 2017 Oct 17;12(10):e0186576. doi: 10.1371/journal.pone.0186576 (PMC5645111; doi:10.1371/journal.pone.0186576)
Supplement: S2 File — (PDF) [file pone.0186576.s004.pdf]

https://cge.cbs.dtu.dk/services/

ResFinder 2.1:

- selected threshold – 95%
- selected minimum length - 60%
- selected type of reads – Illumina paired end reads

Zankari E, Hasman H, Cosentino S, Vestergaard M, Rasmussen S, Lund O, Aarestrup FM, Larsen MV. Identification of acquired antimicrobial resistance genes.J Antimicrob Chemother. 2012 Jul 10.

VirulenceFinder 1.5:

- selected species - E. coli
- selected threshold for %ID – 98%
- selected minimum length - 60%
- selected type of reads – Illumina paired end reads

Joensen KG, Scheutz F, Lund O, Hasman H, Kaas RS, Nielsen EM, Aarestrup FM. Real-time whole-genome sequencing for routine typing, surveillance, and outbreak detection of verotoxigenic Escherichia coli. J. Clin. Micobiol. 2014. 52(5): 1501-1510

MLST 1.8 (MultiLocus Sequence Typing):

- selected MLST configuration - E. coli#1
- selected type of reads – Illumina paired end reads

Larsen MV, Cosentino S, Rasmussen S, Friis C, Hasman H, Marvig RL, Jelsbak L, Sicheritz-Pontén T, Ussery DW, Aarestrup FM and Lund O. Multilocus Sequence Typing of Total Genome Sequenced Bacteria. J. Clin. Micobiol. 2012. 50(4): 1355-1361.

PlasmidFinder 1.3

- selected database - Plasmid – Enterobacteriaceae
- selected threshold for %ID – 98%
- selected type of reads – Illumina paired end reads

Carattoli A, Zankari E, Garcia-Fernandez A, Volby Larsen M, Lund O, Villa L, Aarestrup FM, Hasman H. PlasmidFinder and pMLST: in silico detection and typing of plasmids. Antimicrob. Agents Chemother. 2014. April 28th.

CSI Phylogeny 1.1:

- Uploaded reference genome – VI-2011-01-1292
- Selected min depth at SNP positions – 10x
- Selected min relative depth at SNP positions – 10x
- Selected minimum distance between SNPs (prune) – 10bp
- Selected min SNP quality – 30
- Selected min read mapping quality – 25
- Selected min. Z-score – 1.96
- Used FastTree

Rolf S. Kaas, Pimlapas Leekitcharoenphon, Frank M. Aarestrup, Ole Lund. Solving the Problem of Comparing Whole Bacterial Genomes across Different Sequencing Platforms. PLoS ONE 2014; 9(8): e104984.
