# Supplementary figures and images for: A comparison of extended spectrum β-lactamase producing Escherichia coli from clinical, recreational water and wastewater samples associated in time and location
Source: PLoS One. 2017 Oct 17;12(10):e0186576. doi: 10.1371/journal.pone.0186576 (PMC5645111; doi:10.1371/journal.pone.0186576)

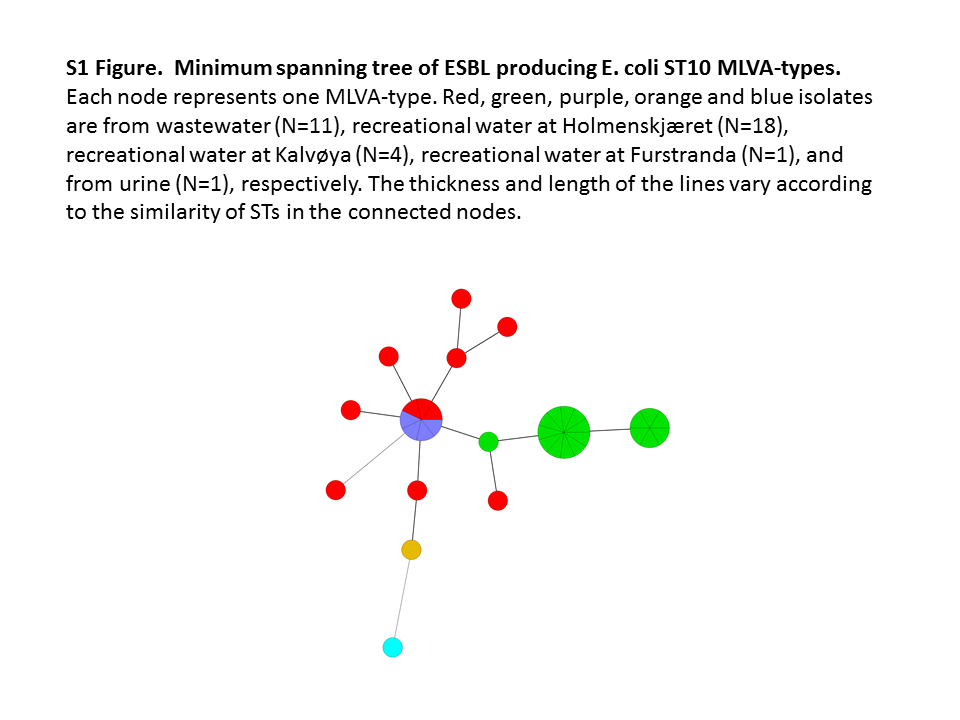

Supplement: S1 Fig — (TIF) [file pone.0186576.s005.tif]
